# Inkjet Etching of Polymers and Its Applications in Organic Electronic Devices

**DOI:** 10.3390/polym9090441

**Published:** 2017-09-11

**Authors:** Wi Hyoung Lee, Yeong Don Park

**Affiliations:** 1 Department of Organic and Nano System Engineering, Konkuk University, Seoul 05029, Korea; 2 Department of Energy and Chemical Engineering, Incheon National University, Incheon 22012, Korea

**Keywords:** inkjet, etching, organic electronic device, organic transistor, organic semiconductor

## Abstract

Inkjet printing techniques for the etching of polymers and their application to the fabrication of organic electronic devices are reviewed. A mechanism is proposed for the formation of via holes in polymer layers through inkjet printing with solvent, and recent achievements in the fabrication with inkjet etching of various three-dimensional microstructures (i.e., microwells, microgrooves, hexagonal holes, and concave structures) are discussed. In addition, organic electronic devices are presented that use inkjet-etched subtractive patterns as platforms for the selective depositions of an emissive material, a liquid crystal, an organic conductor, an organic insulator, and an organic semiconductor, and as an optical waveguide.

## 1. Introduction

Inkjet printing has received considerable attention because of its potential use in future deposition and patterning technologies by the display and semiconductor industries [1,2,3,4,5,6,7,8]. This technique is a direct-write tool for the deposition of functional materials, so it can replace costly vacuum deposition methods such as sputtering and thermal evaporation. In addition, photolithography is not necessary in inkjet printing processes, thereby enabling maskless and photoresist-free patterning. Furthermore, the use of inkjet printing can reduce waste because droplets with picoliter volumes are printed at the desired position. The advantages of inkjet printing over conventional deposition and patterning tools mean that it can be used in the fabrication of components of organic electronic devices such as organic light emitting diodes (OLEDs) and organic thin-film transistors (OTFTs) [3,9,10,11,12,13,14,15,16,17,18,19,20,21,22,23,24,25].

**Figure 1:**
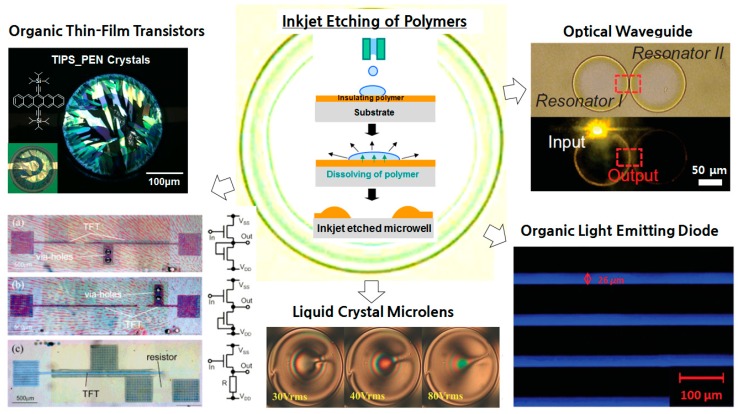
The applications of the inkjet etching of polymers to various organic electronic devices: an optical waveguide, an organic light emitting diode, a liquid crystal microlens, and organic thin-film transistors [11,28,29,30,31].

Although inkjet printing is generally recognized as an additive tool for the deposition of functional materials on target substrates, it can also be used as a subtractive tool for the removal of very small areas of predeposited films [19,26,27]. As shown in Figure 1, the inkjet printing of a solvent onto a polymer film can etch the polymer, which results in the formation of a ring-like deposit through the coffee-ring effect [28]. This inkjet-based, selective wet etching process has several advantages over conventional dry etching processes: it does not rely on conventional photolithography with masks and photoresists, and contamination of the sample can be greatly reduced. This one-shot removal process is also useful for the fabrication of components in organic electronic devices.

In this review paper, we present various implementations of inkjet etching as a subtractive tool for the fabrication of three-dimensional microstructures. Furthermore, the mechanism for the structural development of subtractive patterns is analyzed by considering the underlying chemistry and physics of the etching process. In the following section, the utilization of inkjet-etched microstructures in organic electronic devices is reviewed. Inkjet-etched microstructures can be used directly as optical waveguides, and the selective deposition of organic materials into inkjet-etched microstructures can used to fabricate the components of organic electronic devices. Recent achievements and future perspectives are discussed.

## 2. Inkjet Etching of Polymers

Inkjet etching can be used to fabricate various types of subtractive patterns. When a single solvent droplet is ejected from an inkjet printer, it collides with the pre-deposited polymer film. Then, the polymer dissolves and the solvent evaporates [5,27]. The outward convective flow in the dissolved polymer solution leads to the redeposition of the polymer at the pinned contact line [11,32]. This process results finally in the formation of a crater-like deposit, as shown in Figure 1. The dimensions of the crater-like deposit are determined by several factors such as the properties of the solvent and the polymer [33]. The partial drying of the first jetted droplet can hinder the consecutive printing of ink when the boiling point of solvent is too low. If the boiling point of the solvent is sufficiently high, this first drop problem at the orifice can be resolved and clogging of the nozzle does not typically occur during the printing of solvents [34]. Nevertheless, the careful selection of the solvent is important to ensure both the dissolution of the polymer and consecutive droplet ejection without the formation of satellites. The jettability window of inkjet printing is typically determined by considering the rheological properties of an ink [35]. The Reynolds number (*R*e), the Weber number (*W*e), and the Capillary number (*C*a) are regarded as governing factors for defining printable windows. The ratio of the *R*e to the square root of the *W*e (a quantity called *Z* number) was first identified as a parameter for defining the jettability range [36]. However, recent studies revealed that the *W*e-*C*a space is better to accurately examine the effects of the viscous and inertial forces [35,37]. For instance, Nallan et al. used mixed solvents with different surface tension and viscosity to control both the *W*e and the *C*a and found the appropriate jettability window for stable jetting without wavelike instability [35].

When a single droplet is insufficient to etch the polymer film, successive dropping of the solvent can remove the polymer film without the formation of residue on the substrate. Further, the consecutive printing of solvent droplets during the gradual movement of the inkjet printer head can lead to the formation of microgroove lines. The line width is typically determined by the volume of the droplets, the processing parameters, the substrate temperature, and the polymer-solvent interaction [32,38,39,40]. Several considerations are crucial to the fabrication of polymer-relief microstructures. First, the solubility of the polymer in the solvent must be appropriate: the inkjet-printed solvent should dissolve the pre-deposited polymer on the timescale of droplet evaporation. Evaporation that is too rapid can lead to the uncontrolled etching of the polymer. Second, the thickness of the pre-deposited polymer plays an important role in the design of the dimensions of the etched polymer microstructure. If the thickness of the polymer layer is not optimized, fingering instability can occur and the resulting patterns are not well defined [41,42]. On the other hand, if the polymer layer is too thick, the solvent does not completely etch the pre-deposited polymer. Third, control of the evaporation rate of the solvent enables the fabrication of well-defined microstructures. Note that the etching of the polymer is generally carried out under atmospheric conditions, so the temperature and humidity should be well-controlled [40].

**Figure 2:**
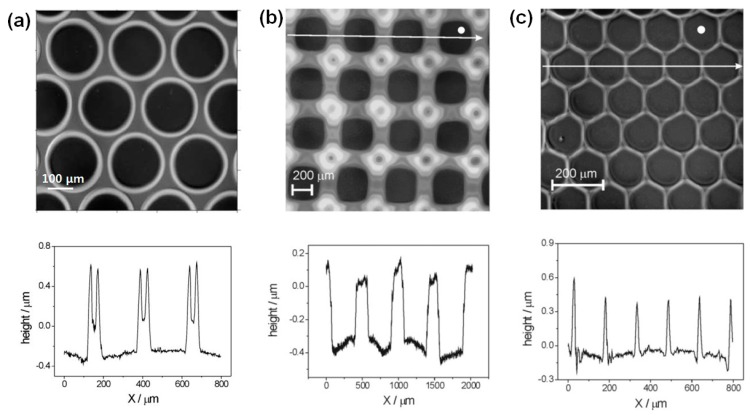
(**a**) Hole array in polystyrene (PS) etched by inkjet printing with isopropyl acetate [33]. Copyright 2007 Royal Society of Chemistry. (**b**) Rectangular holes in PS inkjet-etched with acetophenone droplets. (**c**) Hexagonal holes inkjet-etched with isopropyl acetate droplets. The surface profiles are shown beneath the images [32]. Copyright 2006 Wiley.

Gans et al. examined the formation of various polymer-relief microstructures prepared by inkjet printing solvent onto PS films [31,32]. They used several kinds of solvent and found that the dimensions of the resulting holes or grooves are independent of the choice of solvent (ethyl acetate, isopropyl acetate, *n*-butyl acetate, toluene, anisole, and acetophenone) as long as it is a good solvent for PS [32]. Although the dimensions of the holes or grooves are governed by three factors—the spreading of solvent on the pre-deposited polymer film, the solvent evaporation rate, and the dissolution speed of the polymer film—the dissolution speed of each polymer/solvent pair usually exceeds the solvent evaporation rate. Thus, the solvent evaporation rate is not the governing parameter and the use of solvents with different vapor pressures does not affect the dimensions of the fabricated microstructures. On the other hand, they found that the molecular weight of the PS sample is critical to the formation of subtractive patterns. When they used PS with a high molecular weight (*M*_w_ of 282 kD), the dropping of acetophenone onto the polymer layer did not lead to the formation of such structures. However, the use of PS with low molecular weight (*M*_w_ of 137.4 kD) results in pattern formation, as shown in Figure 2a, which shows an array of holes formed by inkjet printing isopropyl acetate onto pre-deposited PS with a thickness of 180 nm. The height of the ring formed by the coffee staining effect was measured to be approximately 1 μm. They also fabricated patterns of different types by varying the shape of the printing nozzle. When they used a rectangular nozzle, rectangular holes were fabricated (Figure 2b); the use of a hexagonal nozzle resulted in the formation of hexagonal holes (Figure 2c). Interestingly, they found that the distance between the solvent drops affects the shape of the patterns. When the distance between solvent drops was increased for a rectangular nozzle, the shape of the hole changed from rectangular to circular. These findings imply that in inkjet etching the control of the nozzle, i.e., its shape and speed, is important for the control of the shape and dimensions of the resulting pattern.

**Figure 3:**
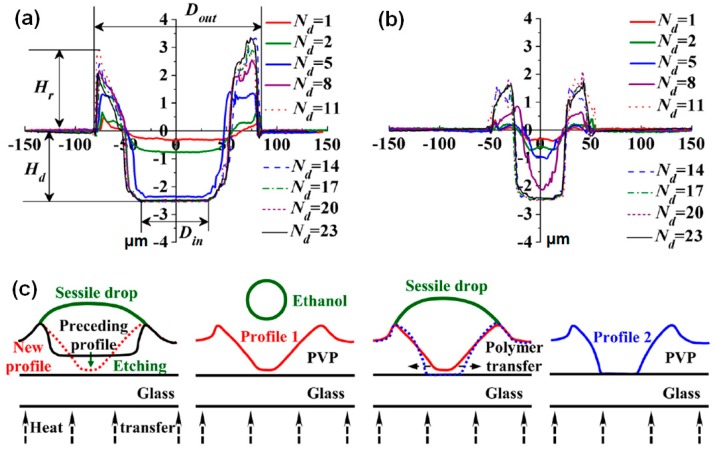
(**a**) Surface profiles obtained after dropping 1 to 23 droplets of ethanol onto poly(4-vinylphenol) (PVP) on a substrate with a temperature of 20 °C. (**b**) Surface profiles of PVP after dropping 1 to 23 droplets of ethanol onto PVP on a substrate at a temperature of 80 °C. (**c**) Schematic diagram of the formation of a hole resulting from the dropping of successive ethanol droplets at a high substrate temperature [40]. Copyright 2013 American Institute of Physics.

Although Gans et al. did not find any relationship between the solvent evaporation rate and the dimensions of the fabricated patterns, Zhang et al. found a clear relationship between them. They controlled the solvent evaporation rate by varying the substrate temperature [40]. Figure 3a,b show comparative plots of via hole size as a function of the number of the drops. PVP, with a thickness of 2.5 μm, and ethanol were used as the polymer film and the etching solvent, respectively. Increasing the substrate temperature enhances both the solvent evaporation rate and the dissolution speed of the polymer film. However, the effects in this system of the increased solvent evaporation rate dominate the effects of the increased dissolution speed. Thus, the etching speed of the PVP film at 80 °C is lower than that of the PVP film at 20 °C. Accordingly, the inner and outer diameters of the holes are reduced for a substrate temperature of 80 °C. Further, the height of the rim that is formed at the elevated temperature is lower because it depends on the volume of the dissolved polymer. Note that the outward convective flow at the pinned contact line results in the movement of the polymer solution to the edge. Figure 3c shows a schematic representation of the formation of a hole through the inkjet printing of successive ethanol drops at a high substrate temperature. This illustration suggests that the reduced polymer transfer at a high substrate temperature is the reason for the reduced diameter of the holes. They also examined the effects of varying the polymer film thickness on the dimensions of the holes fabricated by inkjet etching: as the film thickness increases, the diameter of the holes increases [39]. Further, the number of drops needed for complete etching also increases. Zhang et al. showed that the substrate temperature, film thickness, and polymer surface conditions determine the dimensions of via holes fabricated with inkjet etching [39,40]. Grimaldi et al. examined the effects of varying the substrate temperature and etching solvent on the shape and dimensions of holes fabricated with inkjet etching [43]. They found that increasing the substrate temperature results in holes with a decreased diameter and an increased rim height. Furthermore, a mixed solvent can be adopted to induce a change in the holes from concave to convex. This phenomenon arises because of the resulting change in the flow of the drying polymer solution during the inkjet etching process.

**Figure 4:**
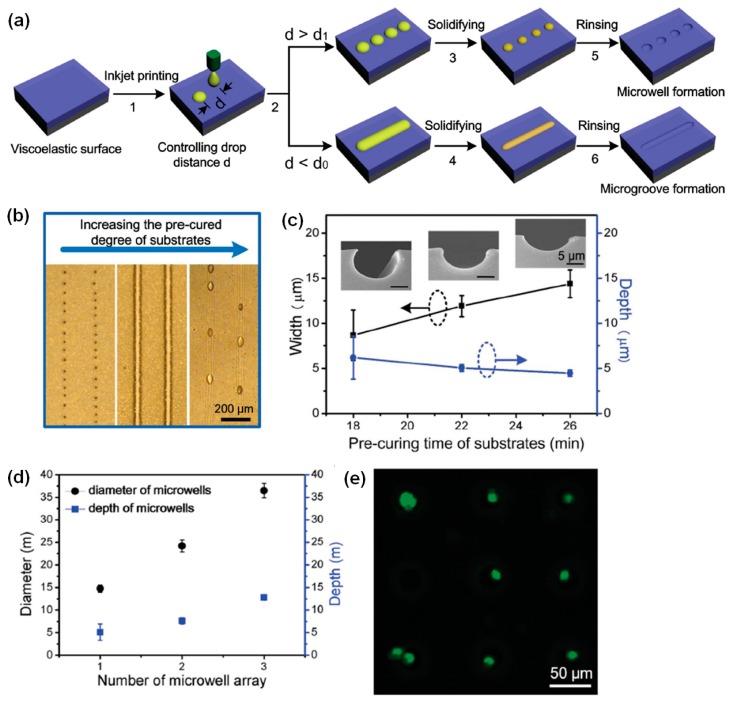
(**a**) The inkjet etching of concave microstructures; (**b**) Optical microscopy images of PDMS films with various degrees of pre-curing after etching with poly acrylic acid (PAA) solution; (**c**) Line width and depth of microgrooves as functions of the polydimethylsiloxane (PDMS) film pre-curing time. The insets show images of the fabricated microgrooves (The scale bars in Figure 4c are 5 μm); (**d**) The dimensions of the microwells used in the trapping of cells; (**e**) Fluorescence image of the microwells with trapped single cells [44]. Copyright 2015 Wiley.

Although the fabrication with inkjet etching of via holes is useful in several research fields, the feature resolution is limited to several tens or hundreds of micrometers. The limited resolution of the inkjet etching process is mainly due to the massive polymer transport resulting from the coffee staining effect. Bao et al. fabricated high-resolution concave microstructures by using the inkjet imprinting technique [44]. They used pre-cured polydimethylsiloxane (PDMS) as a viscoelastic polymer on which the viscoelastic dissipation of solvent occurs [45]. Figure 4a shows the steps in the fabrication of concave microstructures with the inkjet imprinting technique. Microwells or microgrooves can be fabricated by controlling the drop distance: if the drop distance is larger than a critical value, microwells are fabricated; on the other hand, small drop distances lead to the formation of microgrooves. The formation of microwells or microgrooves is dependent on the PDMS pre-curing time. When the pre-curing time is less than 18 min, the ink droplet sinks into the PDMS film, whereas when the pre-curing time is more than 30 min, the ink droplet remains on the PDMS surface. The pattern morphology observations in Figure 4b indicate that the formation of microwells or microgrooves is determined by the PDMS pre-curing time. In addition, the line width is proportional to the pre-curing time, and the line height is inversely proportional to the pre-curing time (Figure 4c). This finding indicates that the spreading of the PAA solution on the PDMS surface is critically affected by the degree of crosslinking in the PDMS film. Microwell arrays with various dimensions were utilized in the trapping of mammalian cells (Figure 4d). Figure 4e shows a fluorescence image of the microwells with trapped single cells. Their results demonstrate that concave microstructures fabricated with inkjet imprinting can be used to efficiently trap single cells for fluorescence imaging.

## 3. Applications in Organic Electronic Devices

Various three-dimensional polymer microstructures (i.e., microwells, microgrooves, hexagonal holes, and concave structures) can easily be fabricated with inkjet etching, and can then be utilized as components in organic electronic devices [20]. In particular, nonlithographic patterning through inkjet etching has the advantage of avoiding the effects resulting from the use of photoresists and developer. Further, the inkjet printing direct-write process enables simple fabrication steps that reduce the quantities of used materials and costs. For instance, inkjet-etched polymer microstructures can be directly utilized as optical waveguides [30]. In addition, they can be used as platforms for the selective deposition of organic materials (i.e., emissive materials, liquid crystals, conductors, insulators, and semiconductors), and thus in high-performance/high-throughput organic electronic devices.

**Figure 5:**
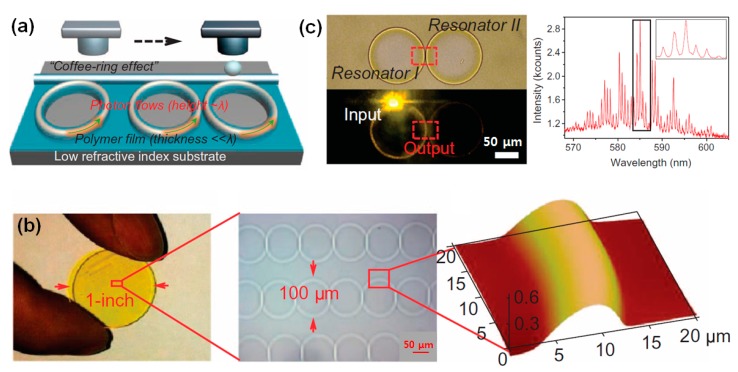
(**a**) Schematic representation of the fabrication of a photonic circuit with inkjet printing. (**b**) Photograph of a photonic circuit with a uniform pattern. An optical microscopy image and an atomic force microscopy (AFM) image of the photonic circuit are shown in the middle and right hand columns, respectively. (**c**) Optical microscopy image of coupled resonators with two rings at a distance of 500 nm and the corresponding spectrum [30]. Copyright 2015 American Association for the Advancement of Science.

The inkjet printing of a solvent onto a polymer film results in the formation of a microring; such a microstructure can be directly utilized to locally induce photon flows in an optical waveguide (Figure 5a). Zhang et al. fabricated a photonic circuit with a uniform microring pattern by using inkjet etching (Figure 5b) [30]. A large-scale ring pattern with a uniform ring distance was fabricated on a flexible plastic substrate. This unique optical-resonator structure can efficiently confine photon flow. Figure 5c shows a coupled optical resonator consisting of two rings separated by a distance of 500 nm. The sub-micrometer scale resolution of the ring pattern was achieved by controlling the inkjet etching conditions. The left ring is excited by incident light and the right ring is illuminated as a result of resonator coupling. The spectrum on the right hand side of Figure 5c demonstrates the successful enhancement of modulated resonance as a result of the Vernier effect. This inkjet-etching-based technique is a promising methodology for the fabrication of optical waveguides with microring patterns, and it is fully compatible with plastic substrates and high-resolution/large-scale production.

**Figure 6:**
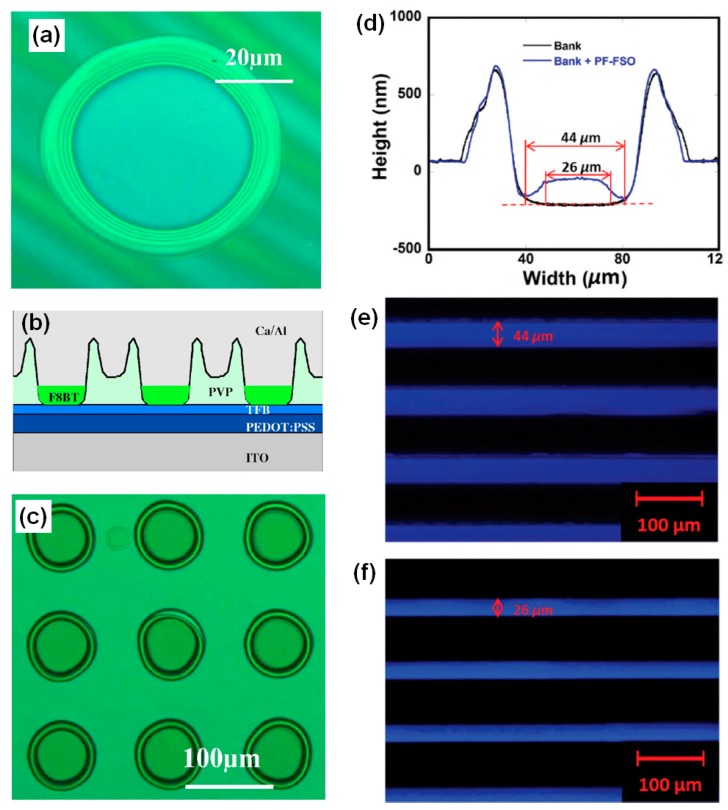
(**a**) A photoluminescence (PL) image of a hole by printing ethanol on a PVP film. (**b**) Schematic showing structure of light emitting diode (LED) with poly(9,9’-dioctylfluorene-*co*-benzothiadiazole) (F8BT) emissive layer. (**c**) PL image of the fabricated LED array [19]. Copyright 2007 American Institute of Physics. (**d**) Surface profiles of inkjet etched CYTOP bank and inkjet printed poly(dibenzothiophene-S,S-dioxide-*co*-9,9-dioctyl-2,7-fluorene) (PF-FSO) film. (**e**) PL and (**f**) electro luminance (EL) images of organic light emitting diodes (OLED) based on PF-FSO emissive layer inkjet printed inside of CYTOP bank [31]. Copyright 2017 Royal Society of Chemistry.

Xia et al. fabricated a regular hole array by inkjet printing ethanol onto a PVP film; Figure 6a shows a photoluminescence (PL) spectrum of the crater-like holes [19]. They then inkjet printed poly(9,9′-dioctylfluorene-*co*-benzothiadiazole) (F8BT) emissive material into the regular hole array, resulting in an integrated OLED consisting of inkjet-patterned PVP banks and an inkjet-printed F8BT emissive material, as shown in Figure 6b. Figure 6c shows a PL image of the fabricated LED array. In their study, the inkjet-etched hole array was found to effectively confine the emissive layer locally during inkjet printing. Similarly, Wang et al. fabricated microgrooves in a fluoropolymer, CYTOP, by performing the successive inkjet printing of solvent [31]. The fabricated CYTOP banks were utilized for the selective deposition with inkjet printing of a poly(dibenzothiophene-S,S-dioxide-*co*-9,9-dioctyl-2,7-fluorene) (PF-FSO) film. The CYTOP banks enable the orthogonal processing of the PF-FSO film because the solvent used in the printing of PF-FSO does not dissolve the CYTOP banks [46]. Figure 6d shows the surface profiles of an inkjet-etched CYTOP bank and the inkjet-printed PF-FSO film. Although PF-FSO could be selectively deposited into the CYTOP bank, it did not cover the edge region. This explained the reason for the difference in PL and electro luminance (EL) spectra, as shown in Figure 6e,f. Patterning of the emissive layer in the OLED was achieved through a nonlithographic process with inkjet etching.

**Figure 7:**
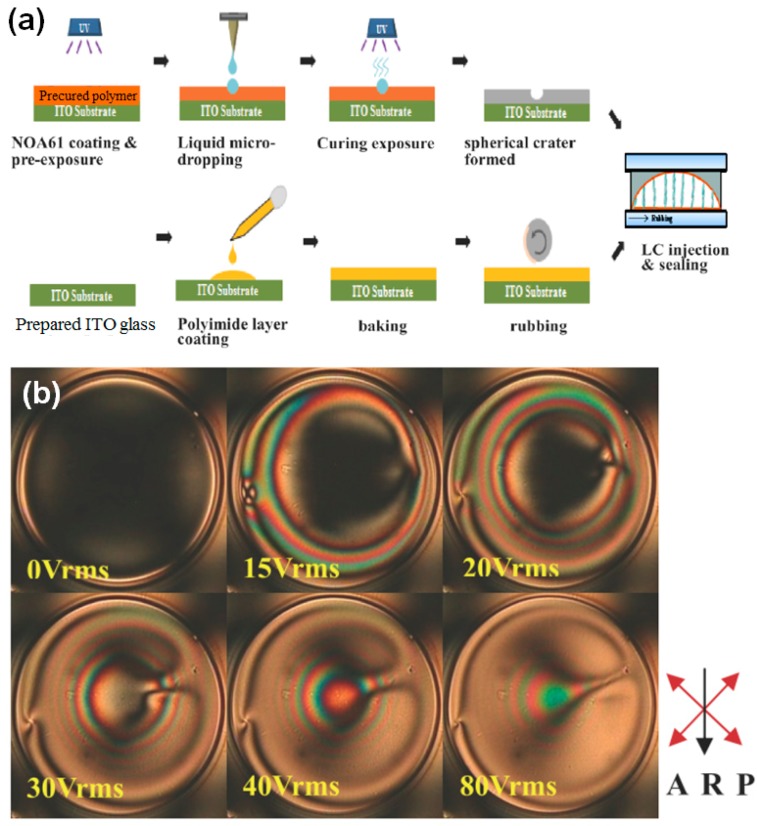
(**a**) Schematic diagram of the steps in the preparation of a liquid crystal microlens. (**b**) Polarized optical microscopy images of the liquid crystal microlens under various applied voltages. R: Rubbing direction; A: Orientation of analyzer; P: Orientation of cross polarizer [29]. Copyright 2013 Optical Society of America.

Hwang et al. used inkjet-etched microwells to fabricate liquid crystal microlenses [29]. Figure 7a shows a schematic diagram of the steps in their method for the preparation of a liquid crystal microlens. UV-curable monomer (NOA61) is partially cured with UV light. Then, deionized water is dropped onto the partially cured NOA61 film and, subsequently, high intensity UV light is utilized both for the complete curing of the NOA61 film and the drying of the water droplet. After UV exposure, a crater-like microwell forms. Here, the two-step UV curing process is the key to the fabrication of the well-defined, crater-like microwell. The injection of liquid crystal into the crater-like microwell finalizes the fabrication of the liquid crystal microlens, and a rubbed polyimide film on the bottom plate is used to align the liquid crystal. The optical properties of the liquid crystal microlens at various applied voltages were measured with polarized optical microscopy (Figure 7b). The color differences indicate that the orientation of the liquid crystals in the microlens can be tuned with an applied bias. The simplicity of their reported fabrication method enables the high-volume production with inkjet etching of liquid crystal microlenses.

**Figure 8:**
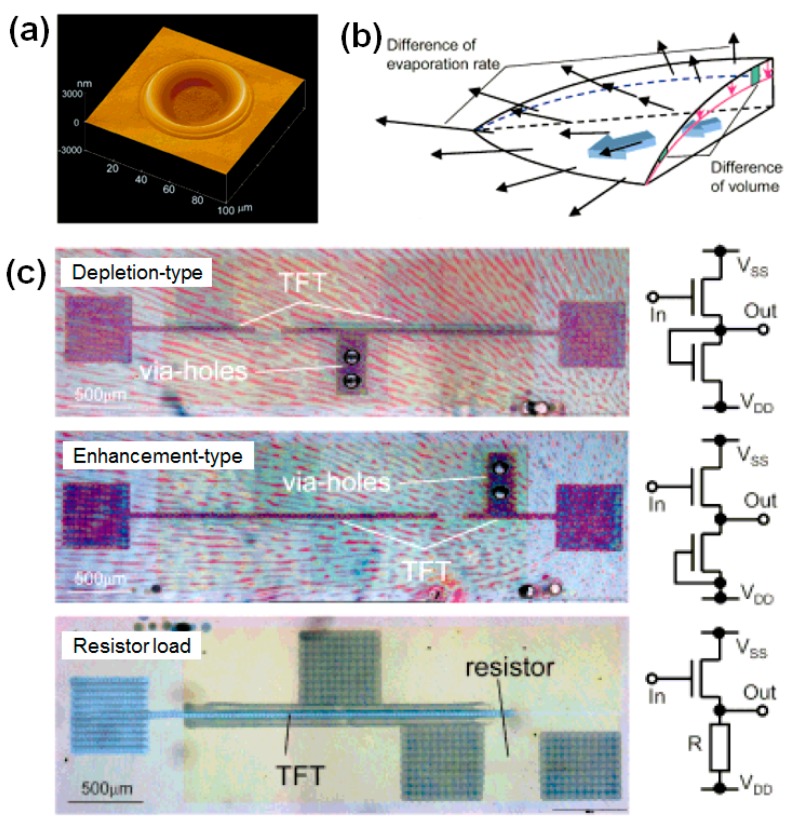
(**a**) AFM image of an inkjet-etched PVP microhole. (**b**) Schematic diagram of the flows in a drying droplet. (**c**) Photographs of three types of inverters (depletion-type, enhancement-type, and resistor load) with poly(3,4-ethylenedioxythiophene) (PEDOT) electrodes connected by via holes in PVP films. The insets on the right show the circuit diagrams of the three types of inverters [11]. Copyright 2001 Wiley.

Inkjet etching is also useful for applications in OTFTs such as interconnection and patterning. Kawase et al. fabricated inkjet-etched via holes to provide interconnections between top and bottom electrodes in OTFTs [11]. Figure 8a shows an atomic force microscopy (AFM) image of an inkjet-etched PVP microhole. As explained in our introduction, the formation of the microhole is driven by the convective flow in the drying polymer solution, which is itself generated by the difference between the solvent evaporation rates of the middle and edge regions (Figure 8b). The PVP dielectric in an OTFT was etched with inkjet printing, and the deposition of a poly(3,4-ethylenedioxythiophene) (PEDOT) electrode through the via hole resulted in the formation of an interconnection between the top and bottom PEDOT electrodes. Kawase et al. fabricated three types of inverters with PEDOT electrodes connected by via holes in PVP films (Figure 8c), and the inkjet-printed, all-polymer circuits were all found to exhibit the required switching capability. Inkjet etching is particularly promising for such applications because via holes can easily be fabricated by inkjet printing the appropriate solvent onto the desired polymer region.

**Figure 9:**
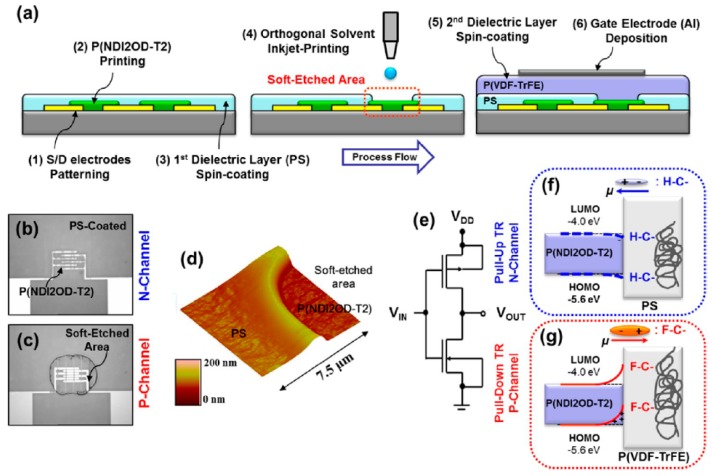
(**a**) Schematic diagram of the fabrication of an inverter with the inkjet-etching technique. Poly([*N*,*N*′-bis(2-octyldodecyl)-naphthalene-1,4,5,8-bis(dicarboximide)-2,6-diyl]-alt-5,5′-(2,2′-bithiophene)) (P(NDI2OD-T2)) was used as the active layer and poly(vinylidenefluoride-trifluoroethylene) (P(VDF-TrFE)) and PS/P(VDF-TrFE) were used as dielectric layers for p-channel and n-channel transistors, respectively. Optical microscopy images of the PS-coated (**b**) and PS-etched (**c**) regions. (**d**) AFM image of a inkjet-etched PS film on a P(NDI2OD-T2) layer, showing the presence of PS microholes. (**e**) Circuit diagram of the complementary inverter. Band diagrams of the semiconductor-dielectric contact regions of the n-channel (**f**) and p-channel (**g**) transistors. Band-bending is determined by the different functional groups in the dielectrics [47]. Copyright 2013 American Chemical Society.

Inkjet etching can be utilized to etch unwanted dielectric materials in OTFTs. Khim et al. used an inkjet-printing-based, soft-etching technique to fabricate high-speed, ambipolar OTFTs. Poly([*N*,*N*′-bis(2-octyldodecyl)-naphthalene-1,4,5,8-bis(dicarboximide)-2,6-diyl]-alt-5,5′-(2,2′-bithiophene)) (P(NDI2OD-T2)) was used as the active layer and poly(vinylidenefluoride-trifluoroethylene) (P(VDF-TrFE)), and PS/P(VDF-TrFE) were used as the dielectric layers of p-channel and n-channel transistors, respectively [47]. Figure 9a shows a schematic diagram of the fabrication of an inverter with a dual gate structure. Inkjet etching was utilized to selectively remove PS and thus to prepare p-channel/top-gate TFTs. *n*-Butyl acetate was used as the etching solvent for PS, which does not damage the P(NDI2OD-T2) film. Subsequent deposition of the P(VDF-TrFE) dielectric onto the selectively etched PS film resulted in the direct contact of the P(VDF-TrFE) film with the P(NDI2OD-T2) film, while PS/P(VDF-TrFE) dual dielectric was fabricated with a view point from the bottom-gate structure. The optical microscopy images in Figure 9b,c show that only the PS region was etched in the p-channel TFTs. The formation of a crater-like deposit in the soft-etched area is evident in Figure 9d. The integration of the p-channel TFT and the n-channel TFT resulted in the fabrication of the integrated circuit in Figure 9e. The use of the P(VDF-TrFE) dielectric increases the number of hole carriers and that of the PS dielectric increases the number of electron carriers. These features are illustrated schematically in Figure 9f,g. P(VDF-TrFE) and PS contain different functional groups, so their dipole moments take up opposing directions, which means that the band-bending of these materials occurs in the opposite directions. Finally, they succeeded in fabricating a ring oscillator with a frequency of 16.7 kHz by employing an inkjet-based etching technique.

**Figure 10:**
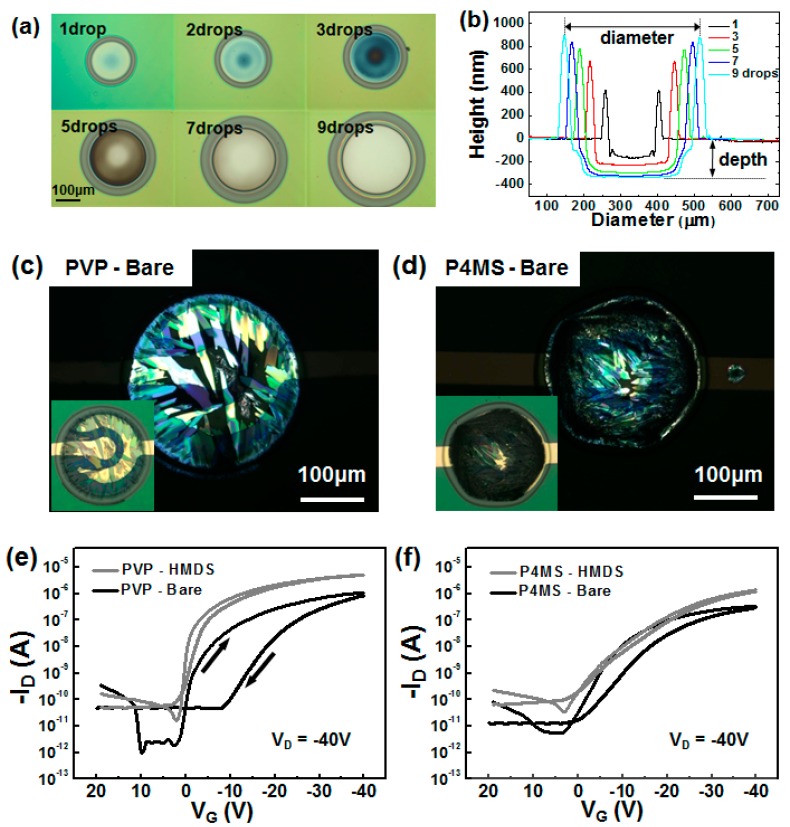
(**a**) Optical microscopy images and (**b**) height profiles of inkjet-etched PVP films for various numbers of printed ethanol droplets. Polarized optical microscopy images of 6,13-bis(triisopropylsilylethynyl)pentacene (TIPS_PEN) crystals that have been inkjet printed on a PVP bank (**c**) and a poly(4-methyl styrene) (P4MS) bank (**d**). Electrical properties of triisopropylsilylethynyl pentacene (TIPS_PEN) thin-film transistor (TFTs) on the PVP bank (**e**) and the P4MS bank (**f**). The term ‘bare’ indicates a SiO_2_ dielectric without surface treatment and ‘HMDS’ indicates a hexamethyldisilazane-treated SiO_2_ dielectric [28]. Copyright 2013 Wiley.

An inkjet-etched microwell can be utilized as a platform for the crystallization of organic semiconductors [15,28]. Kwak et al. fabricated two kinds of microwells with an inkjet-etching technique: a PVP microwell was fabricated through the inkjet printing of ethanol, and a poly(4-methyl styrene) (P4MS) microwell was fabricated through the inkjet printing of toluene [28]. They examined the formation of these microwells by successive dropping of each solvent onto a polymer film (Figure 10a). The diameter and depth of the hole increase with increases in the number of drops. The height of the rim also increases due to the transfer of the polymer material because of the coffee staining effect (Figure 10b). Seven drops of solvent were necessary for the full removal of the polymer layer. The fabricated microwells were crosslinked before the subsequent inkjet printing of 6,13-bis(triisopropylsilylethynyl)pentacene) (TIPS_PEN), which is a well-known organic semiconductor for OTFTs [48,49,50,51,52]. The inkjet-etched microwell banks were then utilized to increase the patterning accuracy of the TIPS_PEN deposits. When the PVP bank was used, highly crystalline TIPS_PEN crystals were formed as a result of contact line pinning and subsequent crystallization at the bank edge (Figure 10c). The printing of the TIPS_PEN solution onto the P4MS bank resulted in small crystallites due to the receding of the contact line and crystallization at the bottom of the substrate (Figure 10d). The TIPS_PEN TFT containing a PVP bank was found to exhibit electrical properties that were superior to those containing a P4MS bank; surface treatment with hexamethyldisilazane led to a further increase in the mobility (Figure 10e,f). The hydrophilic PVP bank was found to induce better contact line pinning and crystal formation on the bank edge. These findings indicate that the surface properties of inkjet-etched microwells are important to their use as platforms for the crystallization of organic semiconductors.

## 4. Conclusions and Outlook

We have reviewed recent reports of the fabrication of subtractive patterns in polymers through inkjet etching. Various types of polymer relief microstructures (i.e., microwells, microgrooves, hexagonal holes, and concave structures) have been fabricated with inkjet etching processes. The pattern shape and dimensions can be controlled by varying the volume of the droplet, the processing parameters, and the substrate temperature, and by modifying the polymer-solvent interaction. The preparation of via holes can be achieved by making use of the coffee staining effect, which is strongly related to the flows in drying polymer solutions that arise during inkjet etching processes. Thus, the in-situ observation of inkjet etching processes can be used to reveal the mechanism underlying the inkjet etching of polymers [53]. In addition, further study of the control of the flows in drying polymer solutions could enable the fabrication of new patterns [3,54]. In order to fully take benefit from inkjet printing technology, the polymer layer needs to be deposited by inkjet printing. In this case, the polymer layer is not flat mainly due to the coffee ring effect. In this regard, inkjet etching behaviors onto this nonflat polymer surface need to be studied for widening use of inkjet printing.

The utilization of inkjet-etched microstructures in organic electronic devices was extensively reviewed. An inkjet-etched microring pattern has been directly utilized as an optical waveguide. The deposition of organic materials (i.e., emissive materials, liquid crystals, organic conductors, organic insulators, and organic semiconductors) onto pre-fabricated microstructures has been used to enhance the performance of organic electronic devices. Patterning of the emissive layer, liquid crystal, or organic semiconductor is feasible because inkjet-etched microstructures can be used as platforms for the selective deposition of organic materials. In addition, inkjet-etched via holes have been used as interconnections between electrodes, and inkjet etching has been utilized to etch unwanted dielectric materials in the preparation of OTFTs. Furthermore, inkjet-etched microwells have been utilized as platforms for the crystallization of organic semiconductors. Inkjet etching is an extremely simple process that does not require photolithography, and so can be used as a subtractive tool for the fabrication of various organic electronic devices. Reducing the feature size of inkjet etching products is vital to the commercialization of this technique in, for example, the display industry [55,56].
